# Changes of antithrombotic prescription in atrial fibrillation patients with acute coronary syndrome or percutaneous coronary intervention and the subsequent impact on long-term outcomes: a longitudinal cohort study

**DOI:** 10.1186/s12959-021-00353-z

**Published:** 2021-12-14

**Authors:** Chiao-Chin Lee, Chiao-Hsiang Chang, Yuan Hung, Chin-Sheng Lin, Shih-Ping Yang, Shu-Meng Cheng, Fan-Han Yu, Wei-Shiang Lin, Wen-Yu Lin

**Affiliations:** 1 Division of Cardiology, Department of Medicine, Tri-Service General Hospital, National Defense Medical Center, Taipei, Taiwan; 2 Division of Cardiology, Department of Internal Medicine Tri-Service General Hospital, National Defense Medical Center, No. 325, Cheng-Gong Road, Section 2, Neihu 114, Taipei, Taiwan

**Keywords:** Antithrombotic, Prescription, Atrial fibrillation, Acute coronary syndrome, Percutaneous coronary intervention, Cohort

## Abstract

**Objectives:**

The choice of optimal antithrombotic therapy in atrial fibrillation (AF) patients with acute coronary syndrome (ACS) or percutaneous coronary intervention (PCI) remains controversial. The aim of this longitudinal cohort study is to investigate the prescribing pattern of antithrombotic regimen in different cohorts and its subsequent impact.

**Setting and design:**

Longitudinal data from the Tri-Service General Hospital-Coronary Heart Disease (TSGH-CHD) registry, between January 2016 and August 2018 was screened.

**Participants and method:**

Patients with prior history of nonvalvular AF, who had ACS presentation or underwent PCI were selected, and these patients were divided into cohort 1 and cohort 2, according to the index date of antithrombotic prescription before and after the PIONEER AF-PCI study.

**Primary and secondary outcomes:**

The primary safety endpoints were composites of major bleeding and/or clinically relevant non-major bleeding. The secondary efficacy endpoints included the occurrence of all-cause mortality, stroke/systemic embolization, nonfatal myocardial infarction (MI), and >30-days coronary revascularization.

**Results:**

A total of 121 patients were included into analysis (cohort 1=35; cohort 2=86). Comparing with cohort 1, the prescription rate of triple antithrombotic therapy (TAT) increased from 17.1 to 38.4%, especially the regimen with dual antiplatelet therapy (DAPT) plus low-dose non-vitamin-K dependent oral anticoagulation (NOAC). However, the prescription rate of dual antithrombotic therapy (DAT) decreased (14.3–10.5%), as well as the prescription rate of DAPT (68.6–51.2%). These changes of antithrombotic prescription across different cohorts were not associated with risk of adverse safety (HR= 0.87; 95% CI, 0.42-1.80, *p*=0.710) and efficacy outcomes (HR=0.96; 95% CI, 0.40-2.32, *p*=0.930).

**Conclusions:**

Entering the NOAC era, the prescription of TAT increased alongside the decrease in DAT. As the prescription rate of DAPT without anticoagulation remained high, future efforts are mandatory to improve the implementation of guidelines and clinical practice.

**Supplementary Information:**

The online version contains supplementary material available at 10.1186/s12959-021-00353-z.

## Introduction

Atrial fibrillation (AF) is the most common sustained cardiac arrhythmia in the world and is associated with significant symptoms, impaired quality of life, and cardiovascular morbidity and mortality. [1] Moreover, AF shares several common risk factors with coronary artery disease (CAD), such as age, obesity, hypertension, diabetes mellitus, and dyslipidemia, so that the prevalence of CAD in patients with AF is expected to be high, reported ranging from approximate 20-40%[2, 3].

However, the strategy of optimal antithrombotic therapy in patients with AF and concomitant CAD remains challenging. [4, 5] Thromboembolism as a result of AF has a stasis and fibrin drive, whereas atherothrombosis is mainly driven by endothelial plaque rupture, platelet aggregation, and even partially thrombin generation. [6] Clinically, dual antiplatelet therapy (DAPT) with a P2Y_12_ inhibitor plus aspirin were recommended in patients with acute coronary syndrome (ACS) or who underwent percutaneous coronary intervention (PCI) with implantation of stent. [7, 8] Oral anticoagulation (OAC), on the other hand, has been proved to be superior to DAPT for prevention of stroke and systemic thromboembolism in patients with AF. [9] As a consequence, so-called triple antithrombotic therapy (TAT) with DAPT plus an OAC will be considered after ACS or successful PCI in AF patients. Unfortunately, but inevitably, the more aggressive of antithrombotic regimen being used, the higher risk of bleeding. [10, 11].

Entering the non-vitamin-K dependent oral anticoagulation (NOAC) era, PIONEER AF-PCI (Open-Label, Randomized, Controlled, Multicenter Study Exploring Two Treatment Strategies of Rivaroxaban and a Dose-Adjusted Oral Vitamin K Antagonist Treatment Strategy in Subjects with Atrial Fibrillation who Undergo Percutaneous Coronary Intervention) trial, published in December 2016, was one of the first attempts to try to clarify the optimal antithrombotic strategy in patients with AF undergoing PCI with placement of stents. [12] The study provided that dual antithrombotic therapy (DAT), a P2Y_12_ inhibitor plus rivaroxaban 15 mg once daily was associated with a lower rate of significant bleeding related to standard TAT with DAPT plus vitamin-K antagonist (VKA), without compromising the risk of major adverse cardiovascular events and stent thrombosis. Taking advantage of safety, DAT with combination of a P2Y_12_ inhibitor and a NOAC may be an alternative option; or even a favorable choice to balance the risk of ischemic event and bleeding. [13, 14] However, the contemporary real world data of antithrombotic management is limited. In this longitudinal cohort study, we did not intend to represent which antithrombotic regimen is better than others. Instead, the scope of the study is to demonstrate the cohort effects on the changes of antithrombotic prescribing habits and its impact on patients’ outcomes.

## Methods

### Study cohorts

Tri-Service General Hospital-Coronary Heart Disease (TSGH-CHD) registry is a single-center, prospective, and longitudinal cohort database and it was established since 2014. [15] Patients were eligible for enrollment into the registry if they presented with stable angina (SA) or acute coronary syndrome (ACS), including unstable angina (UA), non-ST-segment elevation myocardial infarction (NSTEMI), or ST-segment elevation myocardial infarction (STEMI). All patients were admitted to the hospital and received coronary angiography (CAG) with or without coronary interventional therapy. All patients’ clinical data have thoroughly been reviewed and recorded by a specialized research assistant. Baseline demographic characteristics, medical history, clinical presentation, laboratory parameters, echocardiographic findings, phenotype of coronary arteries, coronary angiography results, interventional procedures and discharge medication are comprehensively evaluated from medical records.

Patients with prior history of nonvalvular AF, who had ACS presentation or had underwent PCI were selected from the TSGH-CHD registry between January 2016 and August 2018. These patients were indicated clinical requirement of concomitant anticoagulation and antiplatelet therapy. In order to analyze the differences of prescribing pattern of antithrombotic regimen before and after the publication of PIONEER AF-PCI study, we divided these eligible patients into two longitudinal cohorts by the index date of antithrombotic prescription. Patients were categorized into cohort 1 if the prescribing date was before the publication of PIONEER AF-PCI study (December 31, 2016) and cohort 2 was defined as the cohort after PIONEER AF-PCI study. The study was ethically approved by the institutional review board (IRB NO. A202005128). This was an observational study that patients were not recruited to receive intervention. Informed consent was not required in the study.

### Stroke and bleeding risks assessment

Stroke risk was assessed with the use of CHA_2_DS_2_-VASc scores, which has been proved to be correlated with the risk of stroke and systemic embolization among patients with nonvalvular AF who are not receiving anticoagulant therapy. [16] The CHA_2_DS_2_-VASc scores ranges from 0 to 9 and represents the sum of points for the following conditions: congestive heart failure, hypertension, diabetes mellitus, vascular diseases, age of 65-74 years (1 points for each), and age ≥75 years, prior stroke or transient ischemic attack (2 points for each). The clinical utility of HAS-BLED score has been described before and it was used for bleeding risk assessment in our cohort study. [17] The HAS-BLED score ranges from 0 to 9 and represents the sum of points for the conditions: hypertension, abnormal renal function (dialysis, kidney transplantation, or ≥creatinine 2.3 mg/dL), abnormal liver function (aspartate aminotransferase or alanine aminotransferase more than 3-fold the upper limit of normal or bilirubin more than 2-fold the upper limit of normal), previous history of stroke, bleeding history or tendency, labile international normalized ratio (INR), elderly with age >65 years, concomitant usage of drugs of antiplatelet agents or non-steroid anti-inflammatory drug (NSAID), and excess alcohol intake. The higher HAS-BLED score reflects the higher risk of bleeding.

### Antithrombotic regimen

Clinical decisions regarding prescribing antithrombotic regimen were made by the attending physicians after assessing patients’ characteristics, clinical thrombotic and bleeding risk. The antithrombotic regimens were categorized into three major patterns: triple antithrombotic therapy (TAT), DAPT, and dual antithrombotic therapy (DAT). The composition of TAT included DAPT plus VKA, DAPT plus full-dose NOAC, and DAPT plus low-dose NOAC. The composition of DAT included single antiplatelet therapy (SAPT) plus VKA, SAPT plus full-dose NOAC and SAPT plus low-dose NOAC. The full-dose NOAC indicated 50 mg twice daily for dabigatran; 15 mg daily for rivaroxaban; 5 mg twice daily for apixaban; and 60 mg daily for edoxaban. However, the low-dose NOAC indicated 110 mg twice daily for dabigatran; 10 mg daily for rivaroxaban; 2.5 mg twice daily for apixaban; and 30 mg daily for edoxaban.

### Follow-up and study outcomes

The primary safety endpoint of the study was occurrence of major or clinically relevant non-major bleeding as defined by the International Society on Thrombosis and Hemostasis (ISTH). The definition of major bleeding in non-surgical patients was (1) fetal bleeding and/or (2) symptomatic bleeding in a critical organ (e.g. intracranial, intraspinal, intraocular, retroperitoneal, intraarticular, pericardial, or intramuscular with compartment syndrome) and/or (3) bleeding causing a fall in hemoglobin level of 2 g/dL or more, or leading to transfusion at least 2 units of whole blood or red cells. The clinically relevant non-major bleeding was defined as an acute or subacute clinically over bleed that leads to at least one of the following: (1) a hospital admission for bleeding or (2) a physician guided medical or surgical treatment for bleeding, or (3) a change in antithrombotic therapy. The secondary endpoint was efficacy endpoints which included the occurrence of all-cause mortality, stroke/systemic embolization, nonfatal myocardial infarction (MI), and coronary revascularization (>30 days after discharge). All medical records were reviewed carefully, and every patient was followed up to 1.5 years maximally if they did not meet the criteria of our endpoints.

### Statistical analysis

Continuous variables are presented as mean and standard deviation. Categorical variables are presented as the number of patients and the corresponding percentage. The differences between the continuous values were assessed by using an unpaired two-tail Student *t* test or one-way analysis of variance post-hoc Benferroni test for normally distributed continuous variables; Mann-Whitney rank-sum test for skewed variables. Nominal variables were compared with Pearson chi-square test or Fisher exact test. Kaplan-Meier method was used to present cumulative incidence in the two longitudinal cohorts. A Cox proportional regression analysis was conducted to compare the differences of study outcomes between the two longitudinal cohorts, with the results presented as a hazard ratio (HR) with a 95% confidence internal (CI). All statistical analyses were performed with a software package (IBM SPSS Statistics Version 25.0), and differences were considered significant as p value <0.05.

## Results

### Study population and clinical characteristics

**Fig. 1:**
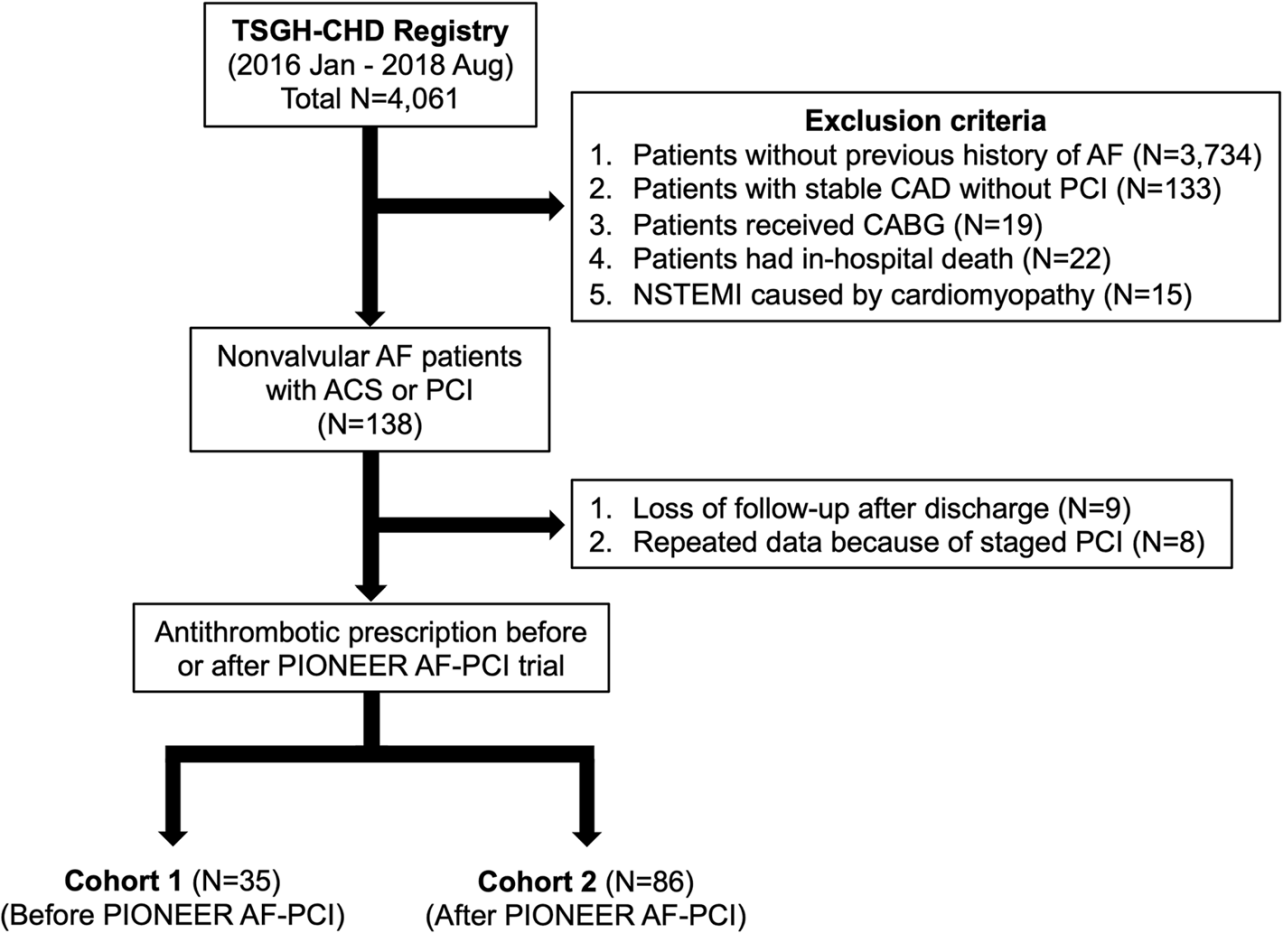
Algorithm of study design. The flow chart demonstrated the selection criteria and patient deposition by the timing of PIONEER AF-PCI study. AF, atrial fibrillation; ACS, acute coronary syndrome; PCI, percutaneous coronary intervention; CAD, coronary artery disease; CABG, coronary artery bypass grafting, NSTEMI, non-ST-segment elevation myocardial infarction

Figure 1 displayed the details of selection criteria and patient deposition. Between January 2016 and August 2018, a total of 4,061 patients were selected retrospectively from the TSGH-CHD registry. After comprehensive assessment, 121 patients with prior nonvalvular AF with ACS or PCI were included into analysis. Among these, 35 patients were categorized into cohort 1 in which the index date of prescribing antithrombotic regimen was before the publication of PIONEER AF-PCI study; and 86 patients were categorized into cohort 2.

**Fig. 2:**
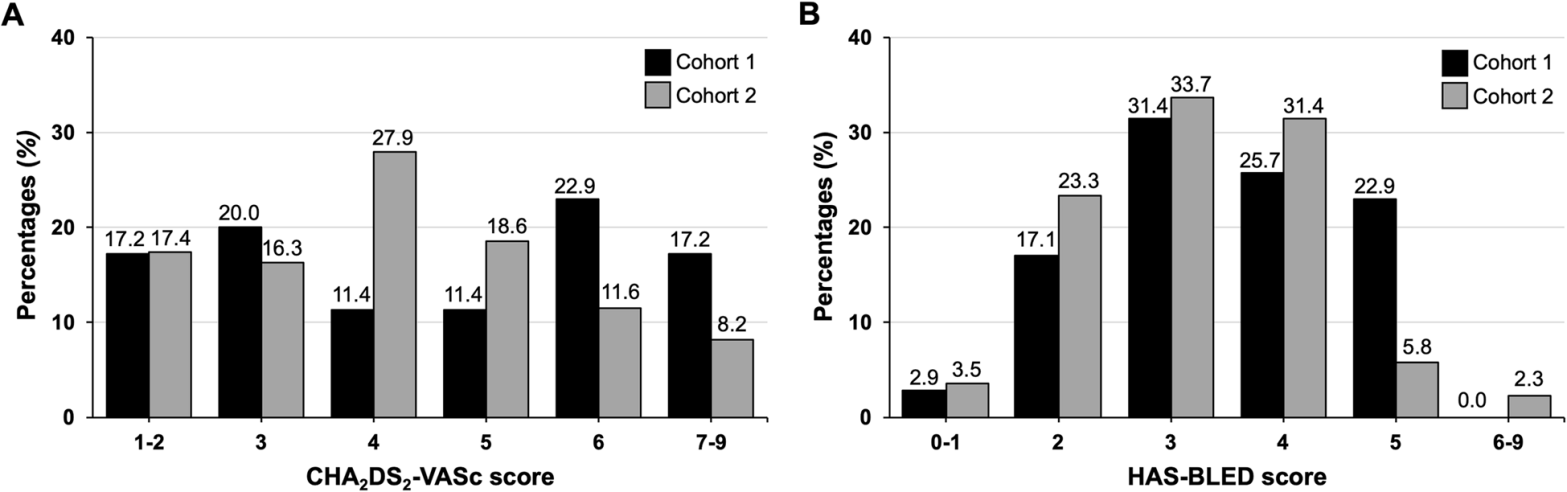
The distribution of CHA_2_DS_2_-VASc and HAS-BLED scores in the two longitudinal cohorts. The detailed information of **(A)** CHA_2_DS_2_-VASc and **(B)** HAS-BLED score were presented. The mean values of CHA_2_DS_2_-VASc and HAS-BLED scores were similar between the two cohorts

Table 1 demonstrated the characteristics of patients in the two cohorts. No differences were observed in age, gender, and underlying comorbidities. The prevalence of uremia, requiring hemodialysis was approximately 20% in both cohorts. Regarding the echocardiographic findings, there were no significant differences in left atrium size and left ventricular systolic function between the two cohorts. The proportion of paroxysmal AF was significantly higher in cohort 1 compared to cohort 2 (68.6% vs. 41.9%, *P*=0.008). The mean CHA_2_DS_2_-VASc (4.5±1.9 vs. 4.1±1.7) and HAS-BLED (3.5±1.1 vs. 3.2±1.1) scores were similar between cohort 1 and cohort 2. Figure 2 demonstrated the detailed distribution of CHA_2_DS_2_-VASc and HAS-BLED scores in the two cohorts. Based on the similar mean CHA_2_DS_2_-VASc score, patients in cohort 1 had more proportion of CHA_2_DS_2_-VASc score of over 5 than cohort 2. On the contrary, patients in the cohort 2 had higher proportion of CHA_2_DS_2_-VASc score of 4 and 5. Regarding the HAS-BLED score, patients in cohort 1 had higher proportion of HAS-BLED score of 5 than patients in cohort 2.

**Table 1:** Comparison of clinical characteristics between two longitudinal cohorts

Characteristics	Overall population (*N*=121)		P value
	**Cohort 1 (***N***=35)**	**Cohort 2 (***N***=86)**	
Baseline characteristics			
Age (years)	71.7±11.4	73.6±12.3	0.418
Male, N (%)	25 (71.4)	60 (69.8)	0.856
Hypertension, N (%)	32 (91.4)	73 (84.9)	0.259
Diabetes mellitus, N (%)	20 (57.1)	37 (43.0)	0.158
Dyslipidemia, N (%)	12 (34.3)	32 (37.2)	0.762
Prior MI, N (%)	18 (51.4)	42 (48.8)	0.796
Prior stroke/TIA, N (%)	9 (25.7)	16 (18.6)	0.381
Prior heart failure, N (%)	17 (48.6)	34 (39.5)	0.361
Uremia, N (%)	7 (20.0)	17 (19.8)	0.977
Echocardiographic characteristics			
LAD (mm)	46.59±7.88	44.04±8.25	0.138
LVEF (%)	49.69±14.36	51.67±16.34	0.552
AF characteristics			
Paroxysmal AF, N (%)	24 (68.6)	36 (41.9)	0.008
CHA_2_DS_2_-VASc score	4.5±1.9	4.1±1.7	0.187
HAS-BLED score	3.5±1.1	3.2±1.1	0.182

MI, myocardial infarction; TIA, transient ischemic attack; LAD, left atrium dimension; LVEF, left ventricular ejection fraction; AF, atrial fibrillation

Table 2 showed the clinical characteristics of coronary artery disease in the two cohorts. There were nearly 40% patients presented as acute coronary syndrome (ACS) in both cohorts and almost 90% of patients had received successful stent placement. The left anterior descending (LAD) coronary artery was the most commonly stenosed coronary artery. Despite the recent practical guideline had recommended contemporary drug-eluting stent (DES) as the preferred stent type during management of PCI in AF patients treated with oral anticoagulation, [18] there was only 51.4% of patients received DES placement in cohort 1 and 61.6% in the cohort 2. No significant differences in stent type were observed between the two cohorts.

**Table 2:** Comparison of clinical presentation, coronary lesion characteristics, and stent types between two longitudinal cohorts

Characteristics	Overall population (*N*=121)		P value
	**Cohort 1 (***N***=35)**	**Cohort 2 (***N***=86)**	
Clinical presentation			
ACS presentation, N (%)	13 (37.1)	33 (38.4)	0.899
Coronary lesion characteristics			
LM lesion, N (%)	3 (2.3)	5 (5.7)	0.421
LAD lesion, N (%)	35 (100.0)	77 (89.5)	0.041
LCX lesion, N (%)	24 (68.6)	54 (62.8)	0.547
RCA, N (%)	28 (80.0)	61 (70.9)	0.305
Stent placement, N (%)	30 (85.7)	77 (89.5)	0.544
Stent types			
DES, N (%)*	18 (51.4)	53 (61.6)	0.302
BMS, N (%)	13 (37.1)	23 (26.7)	0.257
BVS, N (%)	1 (2.9)	1 (1.2)	0.497

ACS, acute coronary syndrome; LM, left main coronary artery; LAD, left anterior descending artery; LCX, left circumflex artery; RCA, right coronary artery; DES, drug-eluting stent, BMS, bare-metal stent; BVS, bioresorbable scaffold

* Two patients received both drug-eluting stent and bare-metal stent placement during the same procedure in cohort 1

### Prescribing pattern of antithrombotic regimen

**Fig. 3:**
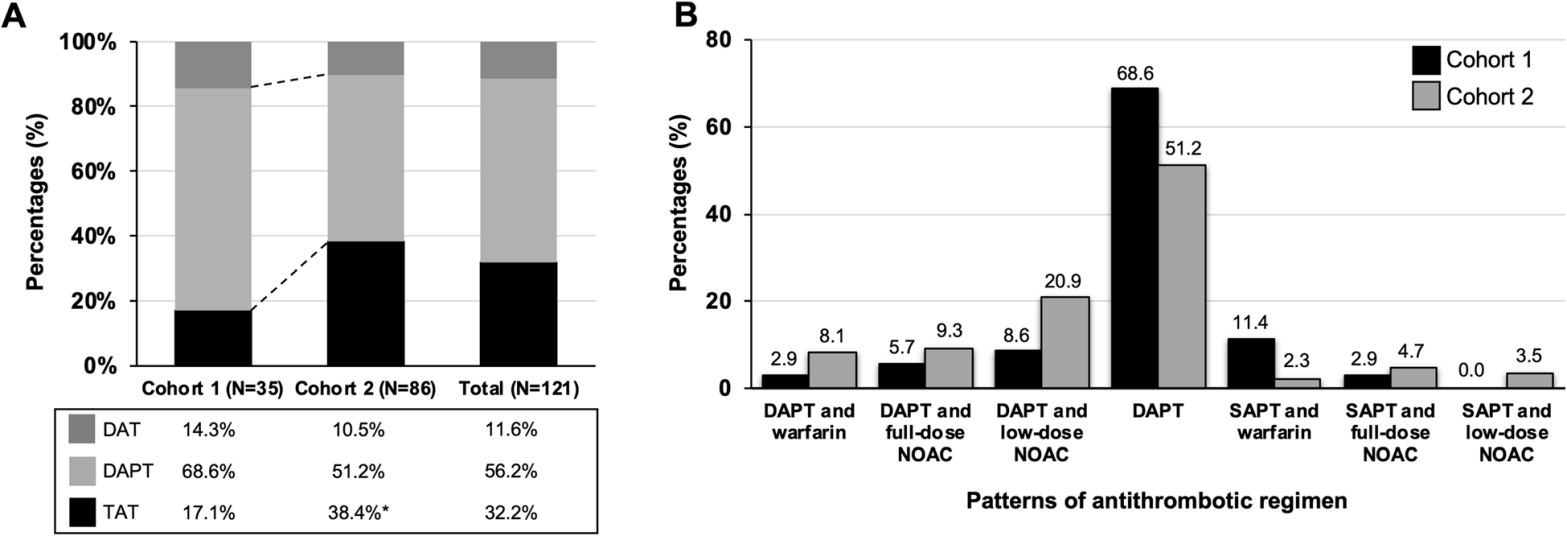
Prescribing patterns of antithrombotic regimen in the two longitudinal cohorts.** A** The bar plot demonstrated the longitudinal changes of prescribing patterns of antithrombotic regimens. **B** The detailed information regarding distribution of all compositions of antithrombotic regimen in the tow cohorts. * indicated the significant difference between the two cohorts

Figure 3 A displayed the percentages of three major prescribing patterns of antithrombotic regimen in the two longitudinal cohorts. Comparing with cohort 1, the percentage of TAT was significantly higher in cohort 2 (17.1% vs. 38.4%, *p*= 0.023). The prescription rate of DAT decreased from 14.3 to 10.5% even despite PIONEER AF-PCI study provided superior safety with DAT, a P2Y_12_ inhibitor plus rivaroxaban (15 mg once daily) over TAT with warfarin. Although the percentage of DAPT decreased from 68.6 to 51.2%, this regimen remained the most common prescription in both cohorts. Comprehensive information regarding all compositions of antithrombotic regimen was illustrated in Fig. 3B. Among the composition of TAT, DAPT plus low-dose NOAC was the most common prescription than DAPT plus warfarin and DAPT plus full-dose NOAC in both cohort 1 and cohort 2. Regarding DAT, the percentage of prescription of SAPT plus a VKA was common (11.4%) in cohort 1, and the SAPT plus a NOAC was more common (8.2%) in the cohort 2.

The prevalence of male gender was much lower in patients received DAT regimen, related to DAPT and TAT (35.7% vs. 77.9% and 69.2%, respectively) (Supplement Table). The prescription of DAT was more common in patients with prior history of stroke or transient ischemic attack. Among patients received DAPT, 32.4% had ESKD requiring hemodialysis that was significantly higher than patients received DAT and TAT. Compared with those received DAT and DAPT, patients received TAT has lower HAS-BLED score (3.5±1.2, 3.4±1.2 vs. 2.9±0.8, respectively).

### Follow-up of study outcomes

**Fig. 4:**
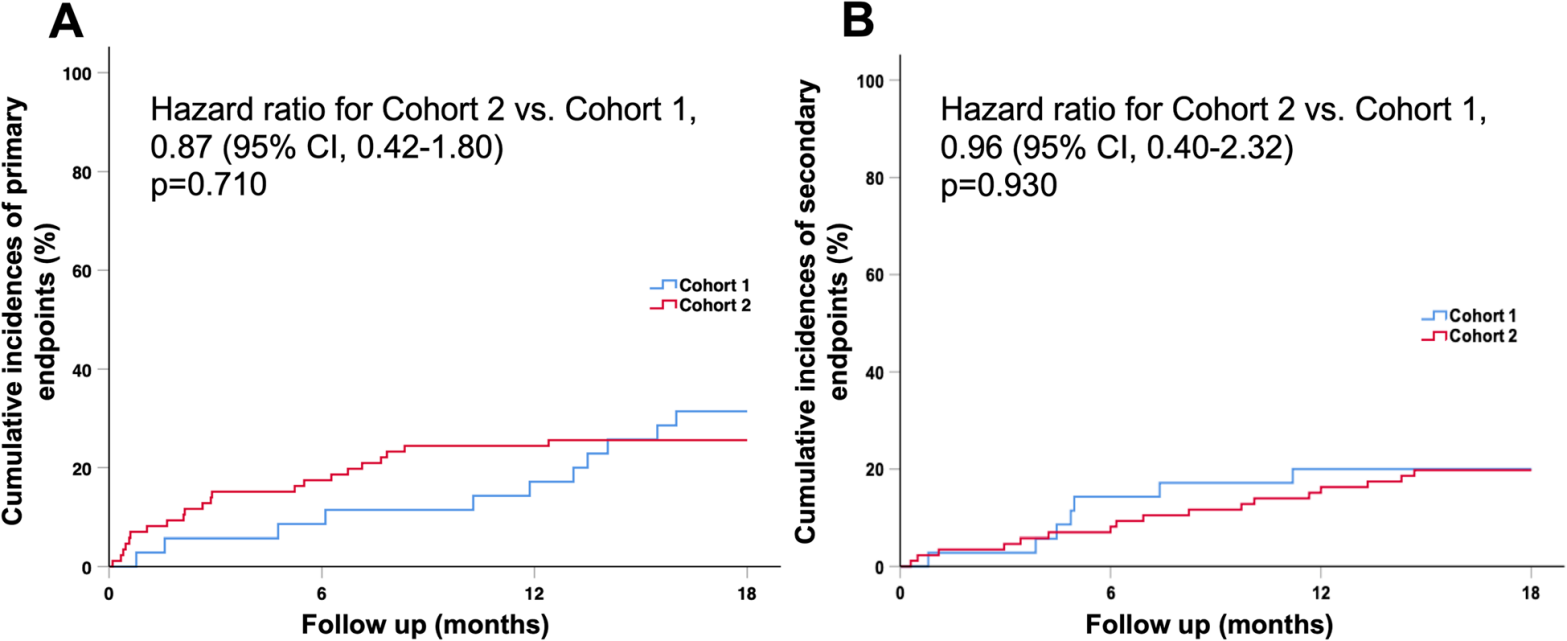
Kaplan-Meier curve of cumulative incidence of study endpoints. The Kaplan-Meier curve of accumulative incidences of **(A)** primary safety endpoints and **(B)** secondary efficacy endpoints showed no significant differences in the two cohorts

Table 3 showed no differences in discharge medication between the two cohorts; including medication of renin-angiotensin system (RAS) blockade, beta-blockade, statin, and antiarrhythmic drugs. During follow-up, 11 (31.4%) patients suffered from primary safety endpoints in cohort 1 and 22 (25.6%) patients in cohort 2 (HR= 0.87; 95% CI, 0.42-1.80, *p*=0.710) (Table 4). There were no significant differences in individual components of the safety endpoints between the two cohorts. Regarding the secondary efficacy endpoints, the incidence was 20.0% in cohort 1 as compared with 19.8% in cohort 2 (HR=0.96; 95% CI, 0.40-2.32, *p*=0.930). The incidence of individual components of the efficacy endpoints were also provided in Table 4. Similarly, no differences in individual components of the efficacy endpoints were observed. Figure 4 demonstrated the Kaplan-Meier curve of cumulative incidence of primary safety endpoints and secondary efficacy endpoints. Although the prescription of antithrombotic regimen changes in different cohorts, the incidence of primary safety endpoints and secondary efficacy endpoints were similar during follow up.

**Table 3:** Comparison of medication at discharge between two longitudinal cohorts

Characteristics	Overall population (*N*=121)		P value
	**Cohort 1 (***N***=35)**	**Cohort 2 (***N***=86)**	
ACEI/ARB, N (%)	13 (37.1)	48 (55.8)	0.063
Aldactone, N (%)	7 (20.0)	23 (26.7)	0.436
Beta-blocker, N (%)	29 (82.9)	65 (75.6)	0.383
Statin, N (%)	21 (60.0)	48 (55.8)	0.673
Digoxin, N (%)	4 (11.4)	9 (10.5)	1.000
Propafenone, N (%)	1 (2.9)	1 (1.2)	0.497
Cordarone, N (%)	7 (20.0)	29 (33.7)	0.134

ACEI, angiotensin converting enzyme inhibitor; ARB, angiotensin II receptor blocker

**Table 4:** Comparison of clinical outcomes between two longitudinal cohorts

Clinical Outcomes	Overall Cohort (*N*=121)		Cohort 2 vs. Cohort 1	P Value
	Cohort 1 (*N*=35)	Cohort 2 (*N*=86)	Hazard ratio (95% CI)	
Primary safety endpoint, N (%)	11 (31.4)	22 (25.6)	0.87 (0.42-1.80)	0.710
Major bleeding, N (%)	10 (28.6)	17 (19.8)	0.74 (0.34-1.62)	0.456
CRNMB, N (%)	1 (2.9)	5 (5.8)	2.16 (0.25-18.45)	0.483
Secondary efficacy endpoint, N (%)	7 (20.0)	17 (19.8)	0.96 (0.40-2.32)	0.930
All-cause death, N (%)	2 (5.7)	8 (9.3)	1.57 (0.33-7.41)	0.567
Stroke/SE, N (%)	1 (2.9)	4 (4.7)	1.60 (0.18-14.28)	0.676
Nonfatal MI, N (%)	2 (5.7)	2 (2.3)	0.39 (0.06-2.79)	0.350
Revascularization, N (%)	2 (5.7)	3 (3.5)	0.60 (0.10-3.59)	0.576

## Discussion

Our study provided the real-world practice of antithrombotic management in AF patients with ACS or PCI in two longitudinal cohorts. Entering the NOAC era, the percentage of DAPT prescription decreased but it remained the most common antithrombotic regimen rather than TAT and DAT. The prescription of TAT, especially low-dose NOAC-based regimen increased, accompanied with decrease of DAT prescription. Moreover, these changes of prescribing pattern of antithrombotic regimen were not associated with long-term risks of clinical bleeding, all-cause mortality, and adverse cardiovascular events.

### Antithrombotic strategy in the NOAC era

A major breakthrough in the antithrombotic management in AF patients requiring anticoagulation has been represented by the introduction of NOACs. [18–21] Moreover, four large clinical trials addressed on the optimal antithrombotic strategy for specific subgroup of AF patients with ACS or PCI requiring concomitant DAPT has been recently generated. [12, 22–24] The PIONEER AF-PCI study, the first one of them, compared three treatment strategies in AF patients after PCI and the study demonstrated the superiority in safety outcomes with DAT (rivaroxaban 15 mg once daily plus a P2Y_12_ inhibitor, mostly clopidogrel) relative to traditional TAT with DAPT plus VKA. [12] The rates of cardiovascular death, myocardial infarction (MI), or stroke were similar between these two antithrombotic regimens. Univocally, other three trials also showed significantly reduced risk of major bleeding in prescribing DAT with a P2Y_12_ inhibitor plus a NOAC when compared with TAT. A recent meta-analysis also suggested combination of a P2Y_12_ inhibitor and a NOAC, without aspirin may be the most favorable treatment option as TAT with DAPT plus VKA may cause more bleeding risk without improvement in antithrombotic efficacy. [25].

However, some key issues should be emphasized. Firstly, the four clinical trials, included AF patients with ACS and/or undergoing PCI were primarily investigating the safety and had no sufficient power to provide definite robust evidence of benefit about ischemic outcome. [26] Secondly, patients with extremely high risk of stent thrombosis were largely under-represented in these trials. In the RE-DUAL PCI trial, there was a numerical trend for increased thrombotic endpoints when treating with SAPT plus dabigatran 100 mg twice daily. [22] In a subgroup analysis of 3498 AF patients underwent PCI with stenting in AUGUST trial, 57 (1.6%) patients developed stent thrombosis over 6 months. The majority of the definite or probable stent thrombosis occurred within 30 days. [27] In addition, according to a comprehensive meta-analysis, Ravi V. et al. suggested the potential increase in the risk of stent thrombosis when choosing DAT in patients with AF and CAD, especially for those at a higher risk of ischemic events. [28] Our longitudinal cohort study showed the prescription of DAT decreased from 14.3 to 10.5%. In cohort 2, only 4.7% of patients received DAT with SAPT plus full-dose NOAC at discharge, like the regimen recommended by clinical trials. This finding suggested that cardiovascular interventionist may hesitate to prescribe DAT at charge because of the unmet need of power in preventing ischemic events of stent thrombosis.

### Current guideline/consensus recommendation

Currently, most of guidelines or consensus recommended TAT as the initial medical strategy for most AF patients with ACS and/or underwent PCI, especially those with high ischemic risk and low bleeding risk. [7, 29–31] In the four clinical trials, the duration of TAT between index PCI and study randomization varied from 1 to 14 days. [12, 22–24] That indicated a short course of TAT after coronary stenting could be desirable. In the 2020 ESC guideline for the diagnosis and management of atrial fibrillation, short course of TAT and early cessation (≤1 week) of aspirin was recommended in AF patients with ACS underwent an uncomplicated PCI. [31] The duration of TAT could be extended up to 1 month when risk of stent thrombosis outweighs the bleeding risk. In the North American expert consensus update focusing on AF patients undergoing PCI, TAT was even recommended only during index hospitalization for most patients. [32] DAT with SAPT plus an OAC should be prescribed immediately after hospital discharge and up to 12 months as a default strategy. One-month of TAT should only be recommended for those at high ischemic/thrombotic risk and low bleeding risk. Our study demonstrated the percentage of TAT prescription at discharge increased significantly from 17.1 to 38.4% in the two longitudinal cohorts, especially the increment of DAPT plus low-dose NOAC (from 8.6 to 20.9%). Nevertheless, the information regarding duration of TAT after discharge was limited in our study. Another point is that only half of our patients with coronary stenting received drug-eluting stent (DES) implantation. Despite the recent practical guideline recommended contemporary DES as the preferred stent type during management of PCI in AF patients treated with oral anticoagulation. [33].

### Under prescribing of anticoagulation in real-world data

One important finding in our study is that the percentage of DAPT prescription decreased from 68.6 to 51.2% as more physicians were aware of the optimal antithrombotic management. In other words, that pointed out approximately half of AF patients with ACS or PCI who required concomitant use of antiplatelet therapy and anticoagulation were prescribed with DAPT only rather than TAT and DAT. This under prescribing of anticoagulation reflected a gap between guideline recommendation and real-world practice.

However, our result is not a unique instance, but has its counterpart. [34] Wang et al. reported 53.3% of patients were prescribed DAPT at discharge in a retrospective cohort study in Taiwan, which included AF patients with a new ACS or PCI from 2008 to 2016. [35] In a Korean nationwide study investigating the 10-year trends of antithrombotic prescription from 2006 to 2015, Park et al. reported the prescription rate of TAT increased gradually (22.7–38.3%) in patients with AF undergoing PCI. [36] However, the prescription rate of DAPT remained high with 60.3% in 2015. Mai et al. reported a retrospective data in Southern China that showed the prescription rate of OACs at discharge for patients with AF and ACS from 2013 to 2018 was only 21.7%.[37] The inadequate implementation of guidelines recommendation were more common in Asia as individuals of Asian ethnicity were considered more vulnerable to anticoagulant-related bleeding, especially using VKA. [38] Among 12,165 Danish population of AF patients hospitalized with MI or PCI between 2001 and 2009, only 3,590 (29.5%) patients received DAPT. [39] Rubboli et al. reported that DAPT was prescribed to 18% of AF patients undergoing PCI and stent implantation in a large European multicenter observational study. [40] Several factors may also contribute the inadequate anticoagulation in our study. The prevalence of baseline HAS-BLED score ≥3 were 80% and 73.2% in cohort 1 and cohort 2, which indicated a relatively higher bleeding risk in our cohort and this situation could discourage physicians to pursue more aggressive antithrombotic strategy. Moreover, the prevalence of uremia was high in both cohorts, with approximately one fifth. We believed that was because Taiwan has the highest prevalence of uremia in the world the the incidence of AF is notably high in uremic patients who requiring hemodialysis. [41] Therefore, uremia, a contraindication to NOAC, could also precipitate the under-prescription of anticoagulation. A meta-analysis suggested the use of VKA for AF may be associated with an unfavorable risk/benefit ratio in patients with ESKD. [42] Therefore, physician would rather not prescribe VKA when treating AF patients with ESKD and our study demonstrated that 92% (22/24) of ESKD patients were treated with DAPT only.

### Study limitations

Some limitations were observed in this study. This is a single-center, observational and non-randomized cohort study and it provides regional data only. Some discrepancies may exist in different areas. Another major limitation is the small sample size which may affect the significance of the results. In addition, not all dosage of NOAC were available in the single medical institution (e.g. Rivaroxaban 20 mg) and this may affect the decision of antithrombotic strategy. The lack of information regarding the duration of antithrombotic treatment after hospital discharge may influenced the patients’ outcome during follow-up. Finally, there was a low prescription rate of guideline-medical therapy, including RAS blockade, beta-blockade, and statin in our study which might also affect the clinical outcome during follow-up.

## Conclusions

In this real-world, longitudinal cohort study, we found that the prescribing pattern of antithrombotic regimen in AF patients with ACS or PCI were changing over time. Entering the NOAC era, the prescription of TAT increased, especially the regimen of DAPT plus low-dose NOAC. Noteworthy, still half of individuals of AF with ACS and/or underwent PCI were treated with DAPT only. We need more efforts to increase the physician awareness and improve the adherence of guidelines for the optimal antithrombotic management of AF patients with ACS or PCI. Finally, our study demonstrated these changes of antithrombotic prescription were not associated with increased risk of clinical bleeding, all-cause mortality, and adverse cardiovascular events.

## Supplementary information

**Additional file 1**

## Data Availability

Not applicable.

## Abbreviations

AF: Atrial fibrillation
CAD: Coronary artery disease
ESKD: End-stage kidney disease
ACS: Acute coronary syndrome
PCI: Percutaneous coronary intervention
CAG: Coronary angiography
SA: Stable angina
UA: Unstable angina
NSTEMI: Non-ST-segment elevation myocardial infarction
STEMI: ST-segment elevation myocardial infarction
DAPT: Dual antiplatelet therapy
SAPT: Single antiplatelet therapy
OAC: Oral anticoagulation
NOAC: Non-vitamin-K dependent oral anticoagulation
VKA: Vitamin K antagonist
TAT: Triple antithrombotic therapy
DAT: Dual antithrombotic therapy
DES: Drug-eluting stent
RAS: Renin-angiotensin system
