# Supplementary material for: Changes of antithrombotic prescription in atrial fibrillation patients with acute coronary syndrome or percutaneous coronary intervention and the subsequent impact on long-term outcomes: a longitudinal cohort study
Source: Thromb J. 2021 Dec 14;19:100. doi: 10.1186/s12959-021-00353-z (PMC8670061; doi:10.1186/s12959-021-00353-z)
Supplement: Supplementary file 1 — Additional file 1 [file 12959_2021_353_MOESM1_ESM.docx]

**Supplement Table.** Comparison of clinical characteristics in the overall cohort, according to the antithrombotic regimens

| Characteristics | Overall Cohort (N=121) | | | P value |
| --- | --- | --- | --- | --- |
|  | DAT (N=14) | DAPT (N=68) | TAT (N=39) |  |
| Age (years) | 77.8±10.1 | 72.6±11.5 | 72.13±13.4 | 0.289 |
| Male, N (%) | 5 (35.7) | 53 (77.9)* | 27 (69.2)* | 0.007 |
| Hypertension, N (%) | 11 (78.6) | 62 (91.2) | 32 (82.1) | 0.256 |
| Diabetes mellitus, N (%) | 4 (28.6) | 32 (47.1) | 21 (53.8) | 0.267 |
| Dyslipidemia, N (%) | 4 (28.6) | 28 (41.2) | 12 (30.8) | 0.455 |
| Prior MI, N (%) | 4 (28.6) | 36 (52.9) | 20 (51.3) | 0.244 |
| Prior stroke/TIA, N (%) | 7 (50) | 10 (14.7)* | 8 (20.5)* | 0.012 |
| Prior heart failure, N (%) | 7 (50) | 23 (33.8) | 21 (53.8)^†^ | 0.107 |
| Uremia, N (%) | 0 (0) | 22 (32.4)* | 2 (5.1)^†^ | <0.001 |
| CHA_2_DS_2_-VASc score | 5.0±1.7 | 4.0±1.7 | 4.3±1.8 | 0.135 |
| HAS-BLED score | 3.5±1.2 | 3.4±1.2 | 2.9±0.8 | 0.061 |

DAT, dual antithrombotic therapy; DAPT, dual antiplatelet therapy; TAT, triple antithrombotic therapy; MI, myocardial infraction; TIA, transient ischemic attack.

* indicated significant difference versus DAT

^†^ indicated significant difference versus DAPT
